# Correlation of Boreholes through Well Logs: Application to the Western Sector of Madrid

**DOI:** 10.3390/s23104718

**Published:** 2023-05-12

**Authors:** Jesús Díaz-Curiel, Lucía Arévalo-Lomas, Bárbara Biosca, María Jesús Miguel, Natalia Caparrini

**Affiliations:** 1 Department of Energy and Fuels, School of Mines and Energy, Universidad Politécnica de Madrid, C/Ríos Rosas 21, 28003 Madrid, Madrid, Spain; lucia.arevalo@upm.es (L.A.-L.); barbara.biosca@upm.es (B.B.); 2 Ministerio de Ciencia e Innovación España, Paseo de la Castellana 162, 28046 Madrid, Madrid, Spain; mjesus.miguel@ciencia.gob.es; 3 Department of Natural Resources and Environmental Engineering, School of Mining Engineering, Universidad de Vigo, C/Maxwell. Campus Lagoas-Marcosende, 36310 Vigo, Pontevedra, Spain; nataliac@uvigo.es

**Keywords:** correlation, borehole, well log, geophysical stretches, multilayer aquifer, groundwater, Madrid siliciclastic basin

## Abstract

This study correlates the results obtained from the resistivity and spontaneous potential well logs in six boreholes for water extraction, located in the multilayer siliciclastic basin in the Madrid region, in the center of the Iberian Peninsula. Given the small lateral continuity that the layers considered in isolation show in this type of multilayer aquifer, geophysical stretches, with their corresponding average lithological assignments, have been established to achieve this objective from the well logs. These stretches allow for mapping the internal lithology in the studied area, obtaining a correlation of greater geological scope than that provided by the correlation between layers. Subsequently, the possible correlation of the lithological stretches selected in each of the boreholes was analyzed, verifying their lateral continuity and establishing an NNW-SSE section in the study area. In this work, the transcendence of the correlation of wells up to great distances (about 8 km in total, and average distance of 1.5 km between wells) is focused on the fact that, if there is a presence of pollutant in certain aquifer stretches in a part of the studied area, overexploitation in the Madrid basin will cause the mobilization of this pollutant to the whole basin, with a possible impact on non-polluted areas.

## 1. Introduction

The increasing importance of groundwater is well-known and is widely studied worldwide. In the most arid regions of the planet and specifically in areas of south-eastern Europe, the importance of groundwater cannot be overlooked, and in recent years, increasing resources have been devoted to the study and characterization of aquifers as a strategic resource [1]. The traditional uses of water, in a simplified form, comprise domestic consumption, including its use in industry and agriculture. The latter is of great importance, as one-third of agricultural land in southern Europe is irrigated with groundwater, which has led to the need to introduce regulations for its use [2]. Studies on the quantity and quality of groundwater and the impacts produced have been performed since the 1990s [3], as well as studies related to the overexploitation of groundwater in arid areas [4].

Techniques for the characterization of aquifers help to quantify the resource and understand the water quality, which can determine its intended use. These techniques include hydrogeological studies with pumping tests and geophysical prospecting studies both on the surface and in boreholes through well log measurements [5,6,7,8].

Directly related to water quality, borehole correlation that covers great distances establishes a strategy to avoid the possibly hazardous effects derived from intensive exploitation, as is the case for the dangerously high arsenic contents occurring in the deepest aquifer stretches in a zone of the Madrid Basin [9]. The term “stretch” has been used in order to avoid controversy with other terms such as “units”, which have a different hydrogeological meaning. It refers to a set of layers that is characterized by maintaining, within it, one or more characteristics that are more or less uniform among themselves, differentiating them from the adjacent stretches.

**Figure 1:**
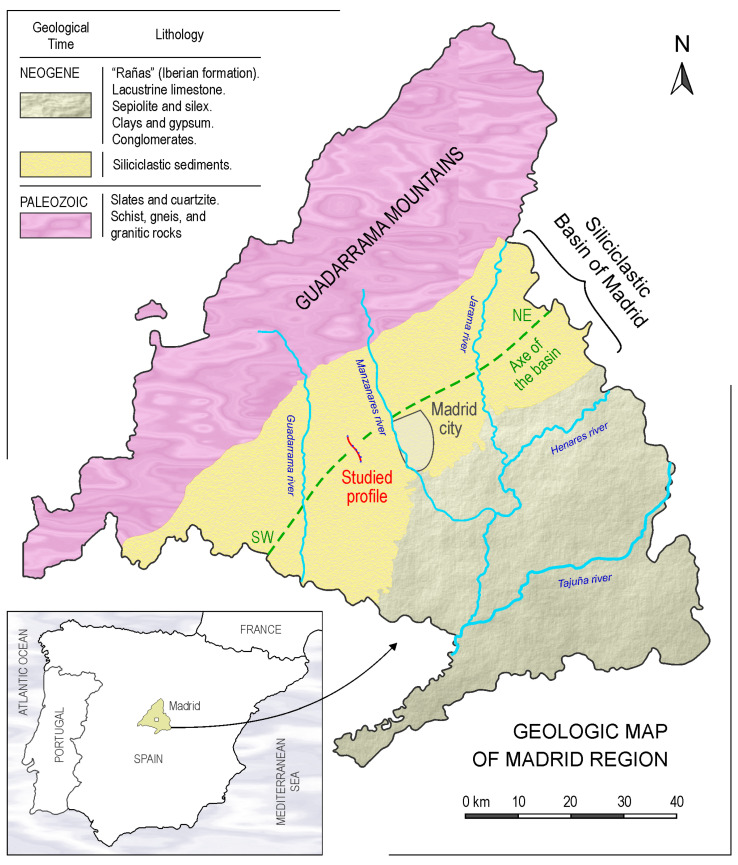
Location of the studied area with the rivers (blue lines), the axis of the Madrid basin (green dashed line) and the studied profile (red line).

The limitations of surface water resources in times of drought and the growing demand for water in the metropolitan area of Madrid, both for domestic and industrial use, have increased interest in groundwater. Thus, the company—Canal de Isabel II (Madrid, Spain)—undertook the drilling and study of a series of wells for groundwater collection located west of the city (Figure 1) in which electrical and natural gamma well logs were obtained.

The initial objective of these studies was to determine the permeable levels of each well for the subsequent laying of screen pipes. However, the lithological correlation between these wells and obtaining a geological section in this area became the final objective in evaluating the potentials of the reservoir as a whole. In multi-layered detrital aquifers, this correlation often lacks sufficient lateral extent to give it sufficient geological scope. Thus, this study proposes the division of the well logs into stretches (series or units) as the best solution to achieve this objective. Moreover, considering that the more permeable geophysical stretches also have a higher vertical transmissivity, the formation water of the permeable layers is much more homogeneous within each stretch.

To this end, the following steps were developed in this study:

- A synthesis of the permeability levels determined by lithological interpretation of the resistivity profiles, gamma ray, and spontaneous potential in the studied wells was conducted.
- Geophysical stretches were established: Layer correlations between well logs taken in relatively close boreholes are possible [10]. These stretches were established in this study as sets of layers with certain similar characteristics, which, on average, differ with respect to the adjacent stretches.
- A correlation of the established stretches was performed to evaluate whether or not they had lateral continuity, obtaining an NNW-SSE section. The scope of this correlation displays a relationship in character, sequence, and position between points of different boreholes in a stratigraphic sense. To obtain a litho-stratigraphic correlation, the concept of belonging to the same lithological unit was added to the analysis.

### Background

Correlations from borehole data provide key data for identifying potential aquifer systems corresponding to large geological units. In large multi-layered siliciclastic continental basins, the correlation between boreholes is more complex than in marine or coastal basins [11,12]. The differences that occur between strata in multi-layered basins are due to differences in size, grain size gradation [13], and clay content, resulting in less repetition of the sequences shown by these strata. Moreover, in this type of basin, changes in layer thickness and the lack of lateral continuity of strata are frequent, even if they are found within the same sedimentary environment. This results in a lack of homogeneity between sedimentary episodes, even leading to the inversion of sedimentary sequences and the appearance of geological discontinuities [14,15]. In these basins, the lateral extension of the layers are typically a few hundred meters; thus, when the distance between boreholes is greater, several characteristics of the well logs associated with the layers are not maintained laterally. These characteristics of unconsolidated detrital environments mean that borehole correlation requires strong support from geological, geochronological, and biostratigraphic information obtained from borehole cuttings [16,17].

The use of well logs for borehole correlation and aquifer modelling has been used by several authors [18,19] with good results. Manual and automatic borehole correlation using well log segmentation has been used in many studies [20,21,22,23,24,25,26]. For years, it has been possible to perform layer correlations between well logs made in relatively close boreholes. Therefore, the division of well logs into stretches, as defined above, is the best solution to achieve this objective, which, to date, has no definitive solution [14,26,27,28,29,30].

One of the main advantages of automating the correlation process is the unification of criteria in which the analyst hardly must intervene. Many of these techniques have used expert systems for many years [31,32,33,34]. However, in this study, the definition of geophysical stretches within the well logs has been considered more manageable for subsequently using the most classical correlation technique that is the cross-correlation, available in most calculation programs. The cross-correlation function between two curves can be defined as the result of multiplying the coincident areas when one moves parallel to the other. This operation produces a new curve that is a function of displacement. This method has been used since the 1960s both in the space domain [35], with good results when there are no large changes in the layer thicknesses, and in the frequency domain [36,37], where the fundamental problems are caused by the absence of layers, although authors such as Srivardhan, 2016 [38] have proposed solutions for this limitation.

The Madrid basin is classified as a heterogeneous and anisotropic unconfined aquifer system [39,40]. However, it is subdivided into great stretches, with some alternating aquifers units separated by non-permeable units (aquitards). This configuration will considerably change the basin model, and the different hydraulic heads obtained in Díaz-Curiel et al., 2022 [41], allow for a very different hydrogeological interpretation of the basin. The subdivision of a large detrital aquifer into stretches with different hydraulic heads [41] also requires analyzing whether the different stretches correspond to aquifers of differentiated water quality.

## 2. Materials and Methods

The two key processes to achieve the expected correlation were the determination of the stretches and the correlative criteria between them, although both processes have been successfully applied in the study of the largest siliciclastic basin in the Iberian Peninsula and the Duero river basin [26]. That basin is characterized by a greater influence of the tectonic cycles that shaped it, whereas, in the Madrid basin, which is a sub-basin of the Tagus River basin, no such influence has been determined. In the study by Díaz-Curiel et al., 2022 [26], the automatic procedures for the processing of the well logs and for the correlation of the established stretches are detailed. In the following, these processes are summarized.

To determine the stretches, two types of statistical criteria were applied to the well logs, defining the stretches from the analysis of the mean values of SP and resistivity on one hand and of their variance on the other hand. Therefore, they would be minimized within the zones and maximized between them. To conduct this process, it is necessary to choose a suitable working window (the thickness over which the log data are analyzed). For this study, a fixed window equal to 14 times the average thickness of the borehole layers was used.

In summary, the procedure for correlating the well logs applied the cross-correlation of the stretches established for the resistivity (SNR) and spontaneous potential (SP) logs. These parameters were chosen because they proved the most representative for this process. To achieve more significant degrees of correlation, these stretches had to be deformed, allowing for the thicknesses of the compared stretches to coincide. For each stretch of the first borehole, the depths of all the recorded points of the other borehole were modified, making the thicknesses of these stretches coincide with that of the compared stretch of the first borehole.

Thus, cross-correlations were made between all stretches of the SNR and SP well logs of the first borehole with those of the same parameters in the second borehole, which consumes a significant amount of time. The degree of correlation between stretches has been obtained by means of the correlation coefficient between two data series, which is given by:

(1) $$Correl\left(X,Y\right)=\frac{\sum \left(x-\bar{x}\right)\left(y-\bar{y}\right)}{\sqrt{\sum {\left(x-\bar{x}\right)}^{2}\sum {\left(y-\bar{y}\right)}^{2}}}$$

where *X* and *Y* are the data series and $\bar{x}$ and $\bar{y}$ are the average value of the respective data series.

**Figure 2:**
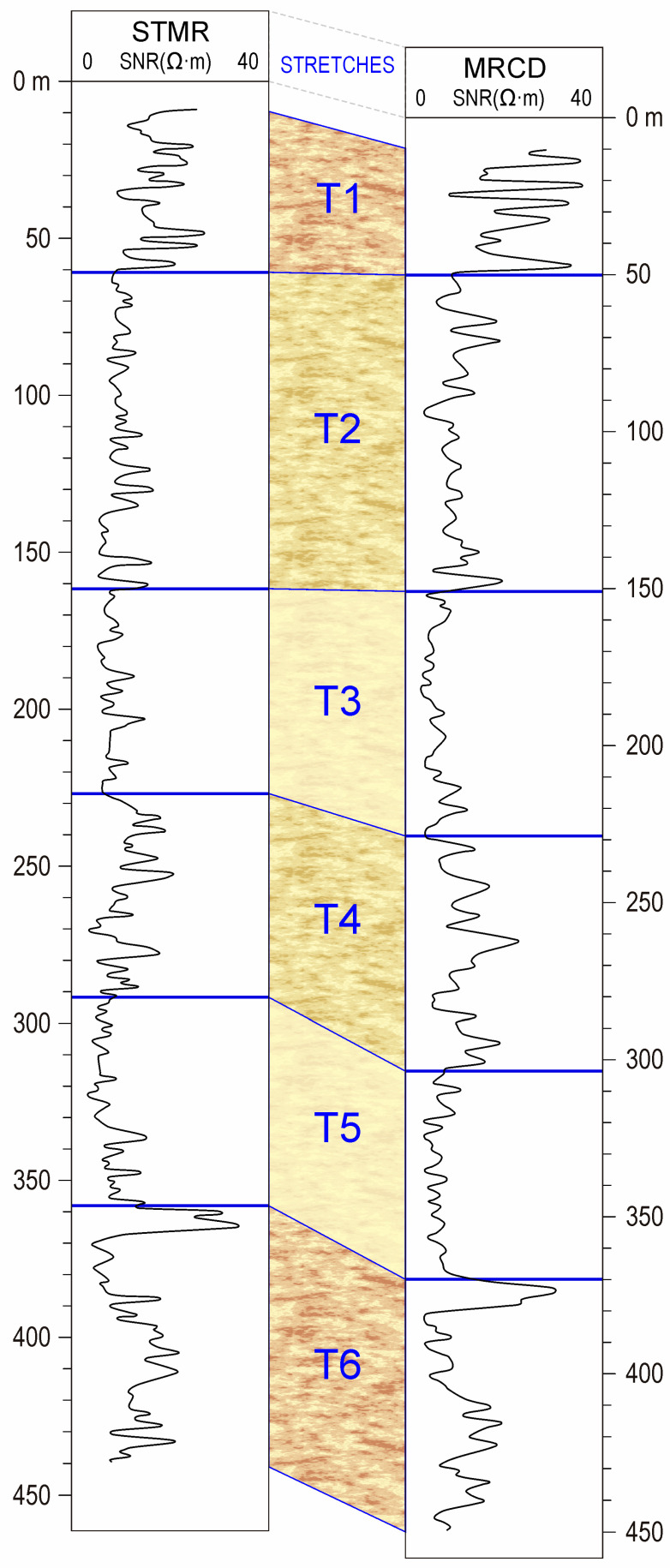
STMR and MRCD boreholes with the established correlation horizons.

The results are presented in a matrix where each cell -*n*- of row -*m*- represents the minimum value of the cross-correlation between stretch -*m*- of the first borehole and stretch -*n*- of the second borehole. In Figure 2, the stretches established in two of the boreholes investigated using the SNR well logs are shown with the determined correlation horizons.

In the case of the correlated boreholes in Figure 2, STMR and MRCD boreholes, the matrix of correlation coefficients between stretches is shown in Table 1.

### 2.1. Geographical and Geological Framework

**Figure 3:**
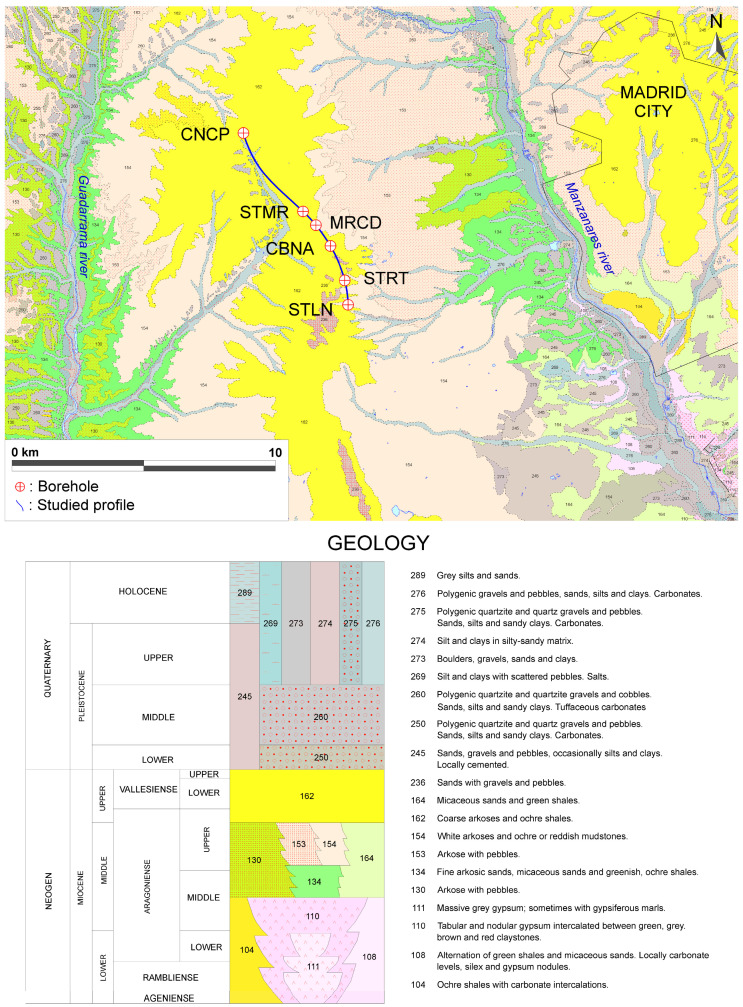
Detailed geology of the area (adapted from IGME, 2021 [42]) and borehole locations.

Geographically, the study area is located in the Tagus River basin of the southern sub-plateau of the Iberian Peninsula (Figure 1). The boreholes analyzed are located in the western sector of the province of Madrid, on the right bank of the Manzanares River, and between the streams of the Trofa River to the north and Butarques River to the south, as shown in Figure 3. The approximate distances between wells are as follows: CNCP-STMR (~3.6 km), STMR-MRCD (~0.8 km), MRCD-CBNA (~1.0 km), CBNA-STRT (~1.5 km), and STRT-STLN (~0.8 km).

Geologically, the study area corresponds to the northwest portion of the Tagus River Trench, composed of Miocene continental sediments. The materials that fill the Tagus Trench are sands, silts, and clays from the erosion of the Central System, formed by Palaeozoic granites, gneisses, slates, and quartzites. These sediments were deposited in an arid continental environment, based on the alluvial fan mechanism, characterized by the complexity of its sedimentary structures. Subsequently, from the Pliocene to the present, an erosive period began which gave rise to a series of alluvial “rañas” (Spanish nomenclature) and terraces.

**Figure 4:**
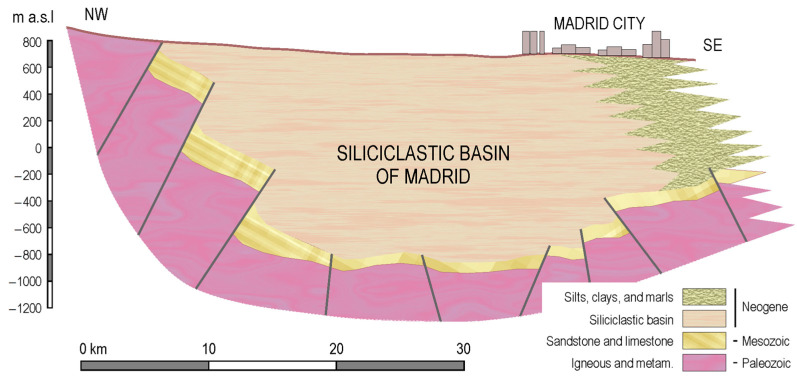
Diagram of the Madrid siliciclastic basin (adapted from Llamas, 1976 [43], and Navarro et al., 1993 [44]).

From a hydrogeological point of view, the aforementioned materials are also found in what is geologically known as the Madrid siliciclastic basin (Figure 4). This aquifer is made of the units of siliciclastic facies, formed by a mass of clays and silts with a variable proportion of sands. On a regional scale, we can speak of an unconfined, anisotropic, and heterogeneous aquifer, in which each unit consists of an irregular alternation of aquifer levels, aquitards, and aquicludes, with one or the other predominating according to the different units. This, in turn, is subdivided into two units—Tosco and Madrid.

1. Tosco Unit:

This unit lies stratigraphically and topographically below the Madrid unit. Its total surface area is approximately 1025 km^2^, but as it underlies the Madrid unit, it outcrops with an extension of 183 km^2^ [45]. 

Based on the data from existing boreholes in this area, a thickness of over 250 m can be assigned to this unit in northern Madrid. In areas affected by erosion, it has a maximum thickness of 120 m and a minimum of 60 m, with the latter coinciding with the vertical extent of the Guadarrama riverbed.

It contains the lateral facies change to the north of the transitional units. This unit, which López-Vera et al., 1977 [46] called the Tosco Formation, is made of medium to fine-grained arkosic sands with silts and clays, coarse arkosic with beds of cobbles and muds, and levels of sepiolite, carbonates, and flints.

2. Madrid Unit:

This unit extends over an area of 816 km^2^, which, when added to the 20 km^2^ below the Quaternary fluvial transports, represents approximately 835 km^2^ [45]. It is composed of coarse-grained arkosic sands, gravels, and clays, which correspond to the last arkosic sedimentary episode observed within the Miocene.

### 2.2. Characteristics of the Boreholes

The boreholes were drilled with reverse circulation rotation, using natural mud as the drilling fluid. Viscosity was maintained at approximately 30 s (in Marsh funnel units, for which conversion to API units, i.e., 10^−2^ cm^2^/s, can be obtained from the relation υ_API_(*T*) = 23.6 + 2.4·υ_MARSH_(*T*) [47]), reaching conductivity values of mud filtrate between 345 and 375 μS/cm in most cases, except in the STRT boreholes, where a value of 1890 μS/cm was reached. Table 2 shows the borehole diameters and study depths for each borehole.

#### Equipment and Probes

The well data logger was a Mount-Sopris 3000 NB, with two vertical scale recorders, with the simultaneous recording capability of four independent parameters and digital speed and depth control. The characteristics of the probes are:

- Combined probe: 0.4 AM (an AM electrode spacing of 0.4 m) normal resistivity, single point resistance, spontaneous potential, and natural gamma (with a 6″ Sodium Iodide crystal length scintillometer detector);
- Temperature probe with PT 100-J sensor with an accuracy of 0.2 °C;
- The radioactive module logged with integration periods of 0.5 and 2 s. The whole unit was calibrated at a USAEC model borehole in Grand Junction, CO, USA.

The average logging speeds were 3.5 m/min for the radioactive and temperature probes, and 6 m/min for the electrical probes to increase the quality of the well logs and reduce the oscillations of the probes.

**Figure 5:**
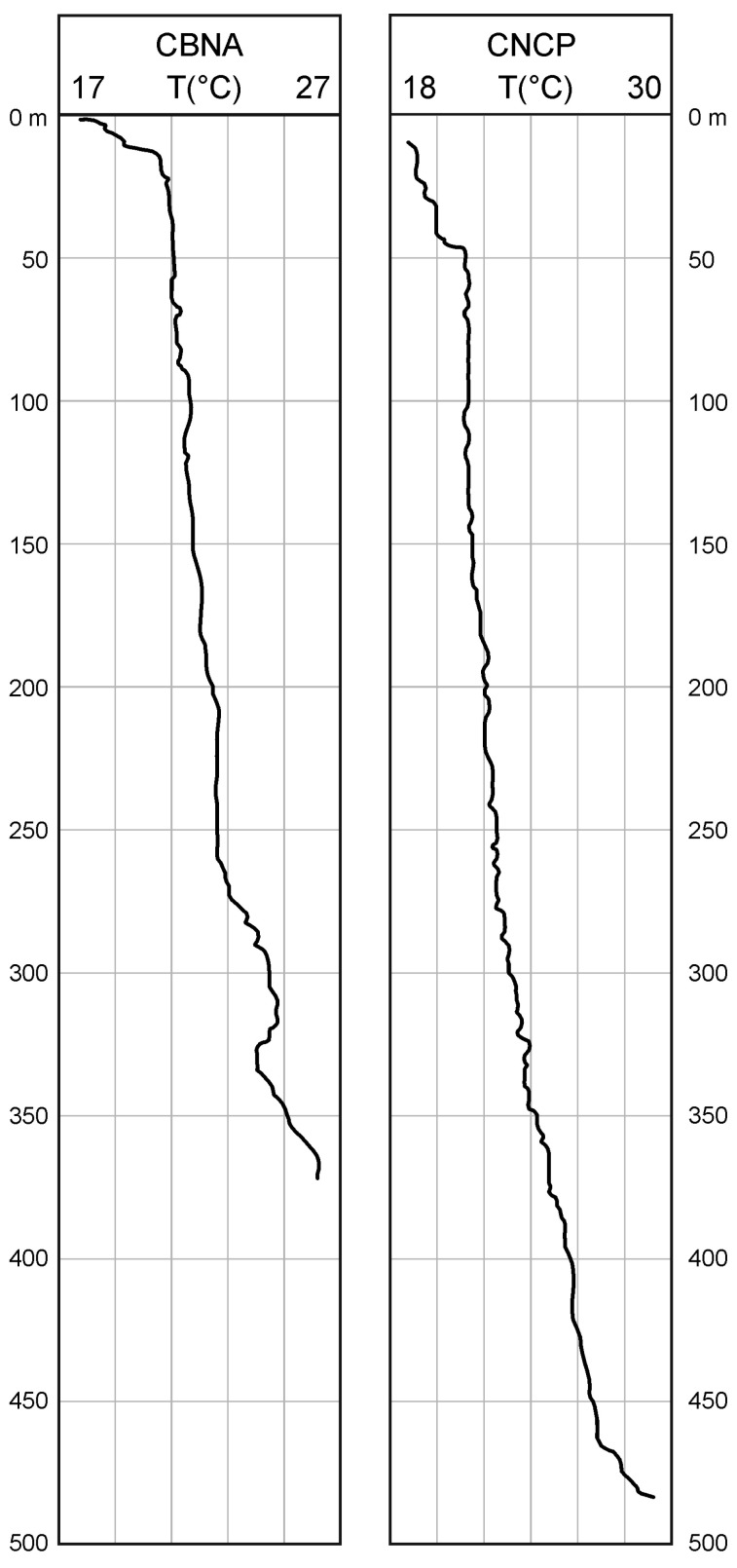
Temperature logs in CBNA and CNCP boreholes.

Temperature logs were taken in only two of the studied wells and were measured to verify that there are no anomalous gradients in the study area. Figure 5 shows the CBNA and CNCP temperature logs in which it is verified that the thermal gradient in the well shows a power growth characteristic of water wells with depths of several hundred meters [47].

## 3. Results

### 3.1. Geophysical Well Logs

The reason for selecting the SP and RNC logs is to show that the geophysical stretches identified with high permeability correspond to aquifers with different hydrochemical properties.

Although the lithological interpretation was carried out using the resistivity, gamma ray, and spontaneous potential well logs in the studied wells, a deficiency found in the gamma ray logs should be pointed out. This deficiency is due to the high borehole diameters and the fact that, in some cases, high density muds were used (being water extraction wells), producing a high invasion diameter which strongly screened the gamma ray logs. In other cases, there was an unjustified time interval between the last mud circulation and the acquisition of the logs, causing a noticeable decantation process.

The lithological analysis of the mud detritus was conducted visually because there was not enough time to perform a detailed sieve grain size analysis to design the screen depths in situ. In this analysis, the predominant grain size of the different samples was first determined by magnifying glass, and then their clay content was estimated with Atterberg limit tests. In this way, the following lithologies were distinguished: coarse sands or gravels, medium-grained sands, fine sands or silts, clayey sands, sandy clays, clays, and loams. This classification does not strictly correspond to the granulometric or mineralogical definitions of these terms, but they are widely used in water boreholes.

#### 3.1.1. Location of Screens

**Figure 6:**
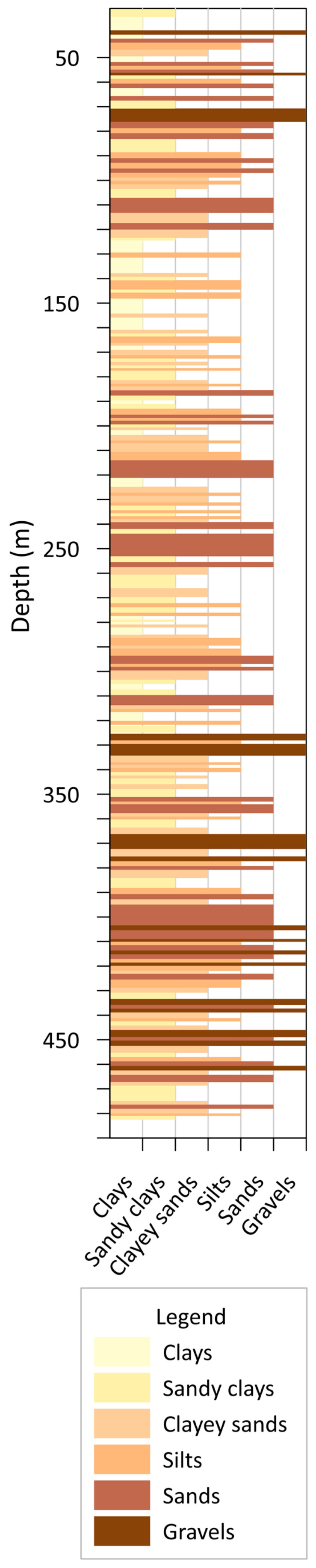
Lithological log interpretation from well logs of CNCP borehole.

In order to decide the location of the filters, we have started from the lithological column obtained from all the logs (supported by the characterization of the samples extracted from the borehole), the permeable levels of each borehole were established (Figure 6 shows the lithological log interpretation in CNCP borehole). Based on this distribution and the constructive characteristics of the borehole (levels, screen lengths, pumping chamber, pipe resistance, welds, etc.), the most interesting intervals for the placement of the screened pipe were determined.

Table 3 shows a summary of the filters recommended in each borehole, with an evaluation of the percentage that they represent of the total depth reached. The screen percentages ranged between 15 and 30%, indicating good characteristics for hydrological use.

In the first meters of the STMR, STLN, and CNCP boreholes, the position of screens was not recommended as the static level is extremely deep. The same was decided for the last meters of the CBNA and STRT boreholes, as they were considered low permeability (clayey) levels.

#### 3.1.2. Determination of Stretches

The geophysical stretches are characterized by the following properties:

- The mean value of the parameters logged, which, because of their relation to porosity and clay content, indicates the dominant lithology.
- The frequency of the curve in each stretch, which refers to the thickness of the different layers as well as their periodicity of occurrence.
- The difference between maximum and minimum values of the well logs, which defines whether the alternation of levels in each stretch corresponds to similar lithologies (e.g., clays and sandy clays) or different ones (e.g., gravels and clays).

For a correct determination of these stretches, as for the lithological assignment, the influence that certain drilling characteristics, such as mud viscosity or borehole diameter, have on the resolution of the geophysical parameters must be known. Similarly, the shifts that certain features, such as mud settling, salinity changes, or borehole diameter variations with depth, produce in the baseline of the spontaneous potential, and gamma ray, as well as in the values of the rest of the parameters, must be discerned. The following is a broad description of the lithological stretches determined in the different boreholes tested.

- CNCP borehole: T1 (0–76 m): Coarse sands with alternating gravels and clayey passes/T2 (76–364 m): Alternation of sandy-clayey or thin sandy levels/T3 (364–485 m): Predominance of coarse sand and gravel levels with considerable clay layers;
- STMR borehole: T1 (0–72 m): Alternating layers of gravels with clayey sands. T2 (72–170 m): Predominantly sandy, with clay intercalations. T3 (170–226 m): Alternating thin layers of fine sands and silts with clay interbeds. T4 (226–300 m): Sand layers approximately 3 m thick with clay intercalations. T5 (300–355 m): Markedly clayey with intercalations of clayey sands and silts. T6 (355–445 m): Alternation of gravels with sands and clay intercalations of variable thickness;
- MRCD borehole: T1 (0–64 m): Alternation of gravels with sandy clays. T2 (64–160 m): Predominantly sands with clay intercalations, which became more abundant towards the base of the stretch. T3 (160–238 m): Thick clay layers with intercalations of clayey sands. T4 (238–306 m): Alternating sandy sands and sandy clays with intercalations of silts and clays. T5 (306–360 m): Markedly clayey with thin intercalations of fine sands and silts. T6 (360–445 m): Alternating gravels and sandy clays;
- CBNA borehole: T1 (0–75 m): Predominantly sandy with considerable levels of gravels and a few passes of clays. T2 (75–178 m): Sandy-clayey stretch with levels of coarse sands of little thickness. T3 (178–260 m): Predominantly clayey. Impermeable stretch. T4 (260–310 m): Levels of coarse sands with intercalations of clayey sands of considerable thickness. T5 (310–375 m): Alternation of clays, fine sands, and silts;
- STRT borehole: T1 (0–82 m): Levels of coarse sands appear with thicknesses of approximately 3 m and clay intercalations. T2 (82–220 m): Alternation of levels of sand and fine sands with others of clays. T3 (220–280 m): Predominantly clayey with a near total absence of permeable levels. T4 (280–315 m): Alternating thin layers of sand with silts and clays;
- STLN borehole: T1 (0–75 m): Coarse sands and gravels with clayey passes. T2 (75–250 m): Sandy-clayey with several thin intercalations of sands. T3 (250–300 m): Predominantly clayey with intercalations of fine sands and silts. T4 (300–430 m): Coarse sand layers of variable thickness along the stretch, with intercalations of clay levels.

### 3.2. Well Log Correlation

#### 3.2.1. Correlation of Geophysical Stretches

Although the Madrid siliciclastic aquifer, on a large scale, has been defined as a free, heterogeneous, and anisotropic aquifer, locally it can be considered as a multilayer aquifer. In this type of aquifer, particularly those corresponding to alluvial fan deposits, when attempting to correlate lithological stretches, extreme precautions must be taken due to the spatial variation of these environments.

**Figure 7:**
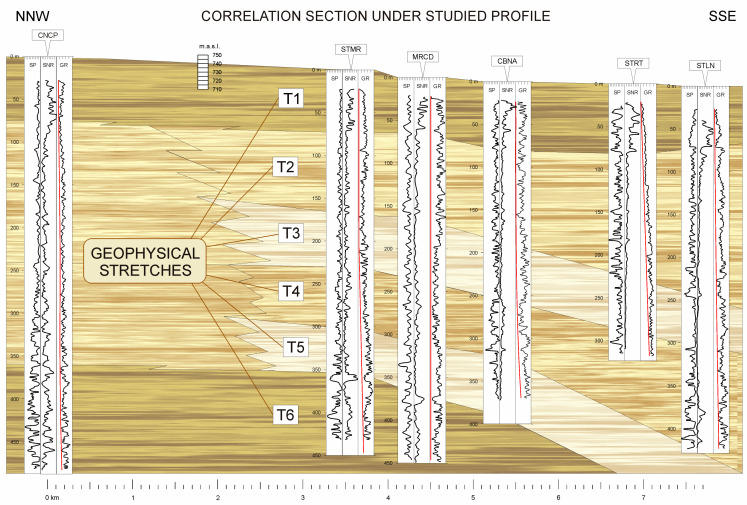
Correlation section together with the established stretches. Red lines represent the variation with depth of the gamma radiation background.

The chosen profile (NNW-SSE) is nearly perpendicular to the source area of the basin, with the consequent limitations of geological correlation, given the sedimentation system that has generated these deposits. Figure 7 shows the normal resistivity (0.4 AM), SP, and gamma ray (together with the lines of variation with the depth of the gamma radiation background) logs for each borehole, as representative of the test results, with the possible correlation horizons.

The continuity of the upper stretch is clearly established. However, we cannot affirm the same for the lower stretches that appear in the CNCP, STMR, and MRCD boreholes, owing to the shallower depth of the CBNA and STRT boreholes. However, the well logs of the STLN borehole indicate that this stretch is still present.

Note the influence of distance on the correlation of well logs in the CNCP borehole, where, being further away from the rest of the boreholes, the subdivision of its second stretch (76–364 m) into four different stretches is not distinguishable, as is the case in the rest of the boreholes.

#### 3.2.2. Column Type

**Figure 8:**
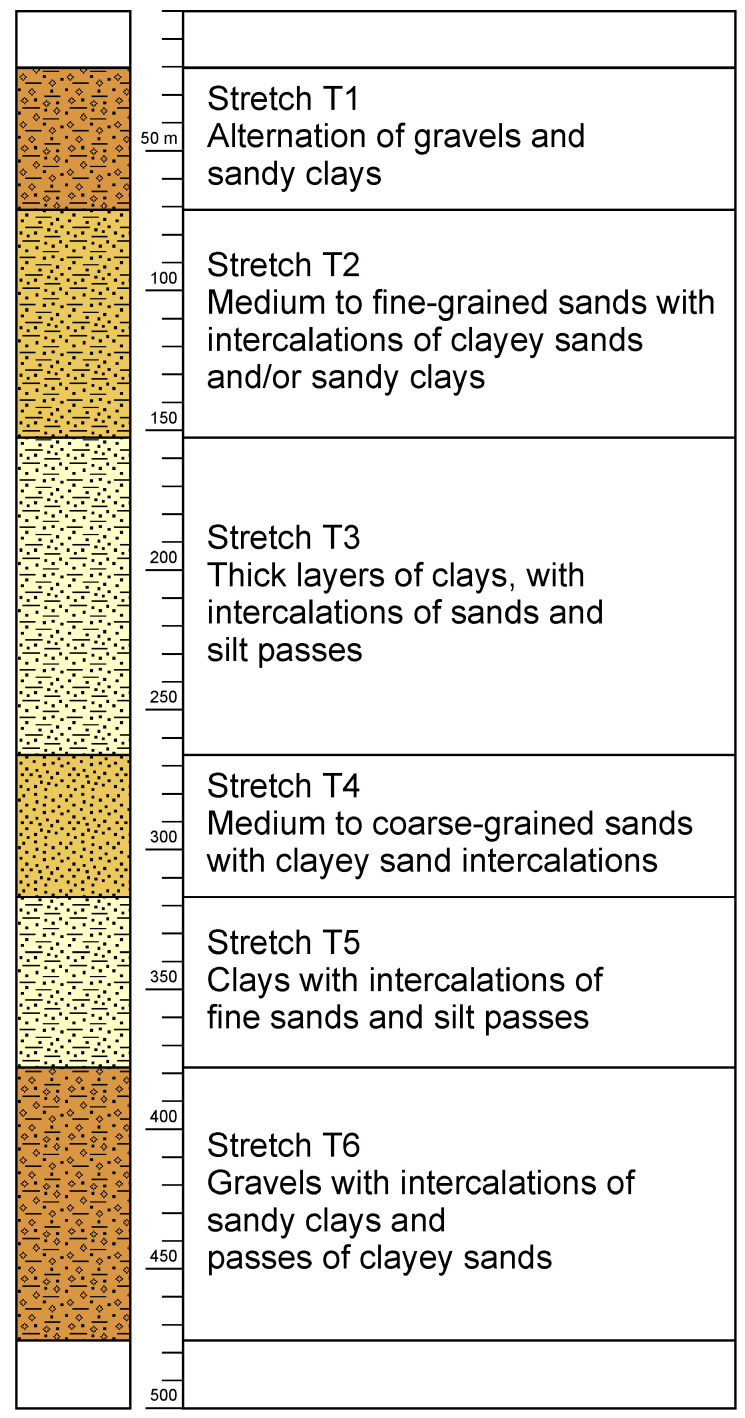
Lithological column type.

Based on the correlation section (Figure 7), it was possible to establish a typical lithological column in this study area. In the following, the stretches of this type of column are described, indicating the minimum and maximum depths where each stretch was found in all boreholes, except for the intermediate stretches in the CNCP borehole. Figure 8 shows a schematic representation of the column described in Table 4.

Finally, in stretches 2, 3, 4, and 5, there is a tendency for the thickness of the clay layers to increase, together with a decrease in the frequency of occurrence of the sandy layers towards the SSE.

## 4. Discussion and Conclusions

The first aspect to discuss is the degree of correlation, assessed by the correlation coefficient between the correlated stretches. These coefficients range between 0.1 and 0.8 in general; therefore, several stretches are correlated with low correlation coefficients (<0.4) owing to the correlation horizons of the other stretches of the two compared boreholes. The values of the correlation coefficients show no apparent relationship with the characteristics of the compared reaches.

Among the parameters assigned to the stretches, on average the highest correlation coefficients occur between stretches with higher variance and mean value. Therefore, sedimentation intervals corresponding to more energetic environments and with higher alternation of lithologies show a higher correlation than stretches formed by more similar lithologies of smaller grain size. In contrast, the frequency has been shown to be less decisive when correlating, confirming one of the limitations of correlation in that domain.

As for the units into which the Madrid siliciclastic basin is divided, according to the literature (IGME, 1992 [38]), the most appropriate concordance would be to state that the Tosco unit is prolonged, at least to the depth of the boreholes analyzed. It also seems that, as a whole, it undergoes a decrease in energy as it ascends in the series.

With respect to water quality, the increase (negative) in spontaneous potential anomalies indicates a gradual increase in salinity. To a lesser extent, this worsening is also observed laterally towards the SE, particularly in the upper part of the Tosco unit (stretch T2). The division of Madrid basin will also allow us to propose a strategy regarding the arsenic propagation throughout and to avoid connecting to a point or zone where the arsenic focus is, as the exploitation of that stretch in different points of the basin will cause the contaminant to move towards those points.

In short, the prior determination of stretch and the comparison between them through the prior variation of their thicknesses allowed the cross-correlation technique to provide effective results. Thus, the correlation established between the geophysical stretches of the studied boreholes leads to the conclusion that the determination of these stretches provides an analysis of a greater extension in multilayer continental basins such as the one studied, in comparison with the correlation between layers. In this respect, one confirmation that can be drawn from the correlation between STMR and MRCD borehole stretches shown in Figure 2 is noticeable—within clearly correlated stretches, the sedimentation sequences of layers or strata do not necessarily hold, even though the correspondence between the series established by these layers is what has traditionally been used for correlation.

This correlation does not imply a correspondence of the same extent between the hydraulic behavior of the wells. This limitation would be explained by the heterogeneity that characterizes this aquifer, with different percentages of fines depending on its location, and by the possible differences in the constructive characteristics of the wells (degree of development, irregularities in the gravel packing, screen location, etc.).

Regarding the general analysis of this area of the basin, the main conclusion is the worsening of the hydraulic characteristics in the boreholes located further to the SSE. These boreholes show an increase in the proportion of clays and silts in this direction, both in the frequency of occurrence of these lithologies and their thickness. This is most evident in the STRT borehole, where more distal facies appear to have been reached. This relatively abrupt variation should be checked in other profiles to establish whether it occurs with respect to the central SW-NE axis of the Madrid siliciclastic basin. It should also be verified whether this variation is related to the basement faults in the Madrid basin.

## Figures and Tables

**Table 1:** Matrix of correlation coefficients (STMR and MRCD boreholes). The shaded background corresponds to the stretches that have been correlated.

		STMR Borehole					
	Stretches	1	2	3	4	5	6
MRCD Borehole	1	0.31	0.02	−0.09	0.25	−0.64	0.35
	2	0.28	−0.12	−0.05	0.09	−0.16	−0.30
	3	−0.09	−0.51	0.19	−0.03	−0.08	0.16
	4	−0.46	−0.25	0.08	0.09	−0.31	−0.04
	5	−0.24	0.01	0.03	−0.09	0.69	−0.10
	6	0.29	0.04	0.11	−0.19	−0.06	0.84

**Table 2:** Borehole diameters and depths.

Borehole	Elevation (m.a.s.l.)	Drilling Diameters (mm)	Depth Ranges (m)	Logging Depth (m)
CNCP	746	660	0–120	485
		445	120–490	
STMR	730	450	0–448	445
MRCD	725	450	0–454	445
CBNA	n/a	660	0–103	375
		445	103–386	
STRT	717	660	0–117	315
		445	117–321	
STLN	715	600	0–430	430

**Table 3:** Recommended screens in each borehole.

Borehole	Pipe Depth (m)	Depth Interval (m)	Number Screens	Total Screens (m)	Screens Percentage (%)
CNCP	483	138–468	50	109.5	22.7
STMR	443	116–443	31	81.0	18.3
MRCD	444	77–450	26	71.5	16.1
CBNA	344	110–320	17	58.5	17.0
STRT	314	49–228	25	65.5	20.9
STLN	423	118–418	20	71.0	16.8

**Table 4:** Description of the stretches.

Stretch	Depth	Description
1	0 to 65–82 m	Clearly permeable stretch. Analyzing the maximum and minimum amplitudes of the curve in this stretch, we observe a considerable difference between them, which represents a strong alternation of gravels with sandy clays. The average thicknesses of the gravel layers are 5–6 m, while the thicknesses of the finer grained layers vary from 2–8 m.
2	From (65–82) to (230–300) m	Less permeable lithologies are evident in this stretch. It is made of an alternation of medium to fine-grained clean sands (with thicknesses ranging from 3–6 m) and intercalations of clayey sands and/or clays of variable thickness.
3	From (160–240) to (200–260) m.	Predominantly low-permeable lithologies. This is a series of layers of clays with thicknesses of approximately 10 m with intercalations of clayey sands and passes of silt.
4	From (230–300) to (300–420) m.	Permeable stretch of medium to coarse-grained clean sands, depending on the borehole analyzed. The thickness of the sand layers ranges from 3–6 m, interspersed by clayey sands.
5	From (300–320) to (355–375) m.	It has been defined as a predominantly clayey stretch, with intercalations of fine sands and silt passes.
6	From 355 m.	Clearly permeable lithologies. It consists of a series of gravel layers between 4–10 m thick with intercalations of sandy clays of varying thickness.

## Data Availability

The data presented in this study are available on request from the corresponding author. The data are not publicly available due to some special reasons.
